# *AML1-ETO*-Related Fusion Circular RNAs Contribute to the Proliferation of Leukemia Cells

**DOI:** 10.3390/ijms24010071

**Published:** 2022-12-21

**Authors:** Ying Wang, Yu Liu, Yingxi Xu, Haiyan Xing, Zheng Tian, Kejing Tang, Qing Rao, Min Wang, Jianxiang Wang

**Affiliations:** 1 State Key Laboratory of Experimental Hematology, Haihe Laboratory of Cell Ecosystem, Institute of Hematology and Blood Diseases Hospital, Chinese Academy of Medical Sciences & Peking Union Medical College, Tianjin 300020, China; 2 National Clinical Research Center for Blood Diseases, Institute of Hematology and Blood Diseases Hospital, Chinese Academy of Medical Sciences & Peking Union Medical College, Tianjin 300020, China

**Keywords:** leukemia, circular RNA, *AML1-ETO*, *RUNX1-RUNX1T1*, proliferation

## Abstract

The *AML1-ETO* (*RUNX1-RUNX1T1)* fusion gene created by the chromosome translocation t(8;21) (q21;q22) is one of the essential contributors to leukemogenesis. Only a few studies in the literature have focused on fusion gene-derived circular RNAs (f-circRNAs). Here, we report several *AML1-ETO*-related fusion circular RNAs (F-CircAEs) in *AML1-ETO*-positive cell lines and primary patient blasts. Functional studies demonstrate that the over-expression of F-CircAE in NIH3T3 cells promotes cell proliferation in vitro and in vivo. F-CircAE expression enhances the colony formation ability of c-Kit^+^ hematopoietic stem and progenitor cells (HSPCs). Meanwhile, the knockdown of endogenous F-CircAEs can inhibit the proliferation and colony formation ability of *AML1-ETO*-positive Kasumi-1 cells. Intriguingly, bioinformatic analysis revealed that the glycolysis pathway is down-regulated in F-CircAE-knockdown Kasumi-1 cells and up-regulated in F-CircAE over-expressed NIH3T3 cells. Further studies show that F-CircAE binds to the glycolytic protein ENO-1, up-regulates the expression level of glycolytic enzymes, and enhances lactate production. In summary, our study demonstrates that F-CircAE may exert biological activities on the growth of *AML1-ETO* leukemia cells by regulating the glycolysis pathway. Determining the role of F-CircAEs in *AML1-ETO* leukemia can lead to great strides in understanding its pathogenesis, thus providing new diagnostic markers and therapeutic targets.

## 1. Introduction

Acute myeloid leukemia (AML) is a genetically diverse hematopoietic malignancy, and chromosomal translocations are associated with particular sub-types of AML. The translocation of t(8;21) (q21;q22) is one of the most common chromosomal rearrangements in AML, which results in the formation of the AML1-ETO (RUNX1-RUNX1T1) fusion protein [1]. It interacts with normal hematopoiesis-related target genes and recruits the transcriptional corepressor complex, eventually leading to hematopoietic differentiation blockage and leukemogenesis [2].

CircRNAs are closed RNA molecules produced by the back-splicing of exons, introns, or both. The reverse complementary Alu repeats flanking the circularized exon have been proposed to be responsible for the biogenesis of circRNAs [3,4]. Furthermore, the exon-skipping event, which leads to an exon-containing lariat precursor, also precedes the circRNAs [5]. First reported more than forty years ago, circRNAs were considered byproducts generated during the normal splicing process, which possessed a less critical biological function [6,7]. Since then, more and more circRNAs have been verified as playing essential roles in diverse biological processes [8,9,10,11,12].

The vital roles that circRNAs play in hematopoietic malignancies are gradually being uncovered. CircRNAs are widely expressed in various blood cell types [13,14,15] and differently expressed between normal and malignant hematopoiesis, indicating that they may be implicated in the occurrence and development of diseases [16,17,18,19]. Increasing evidence has demonstrated that circRNAs derived from fusion genes play vital roles in tumor development. Guarnerio J. et al. first described the fusion-circRNAs (f-circRNAs) derived from tumor-associated chromosomal translocations, such as *PML-RARα, MLL-AF9,* and *EWSR1/FLI1*, and characterized their oncogenic role in hematological malignancies [20]. However, the f-circRNAs derived from *AML1-ETO* have not yet been reported, and little is known about their function and mechanism.

In this paper, we elucidate the characteristics of *AML1-ETO*-related fusion circRNAs (F-CircAEs) in *AML1-ETO* leukemia. Several F-CircAEs (F-CircAE1 to F-CircAE5) were identified from leukemia cell lines and primary AML patients. Over-expression of F-CircAE2 in NIH3T3 (F-CircAE2-NIH3T3) cells resulted in increased proliferation and foci formation. In vivo, F-CircAE2-NIH3T3 cells demonstrated stronger tumorigenicity in immunodeficient mice. On the contrary, the down-regulation of endogenous F-CircAE2 and F-CircAE3 expression in Kasumi-1 cells inhibited cell proliferation and cell cycle progression. Furthermore, F-CircAE2 was confirmed to bind to essential proteins related to glycolysis and promote aerobic glycolysis for leukemia cell growth. Our work demonstrates, for the first time, that *AML1-ETO*-related fusion circRNAs (F-CircAEs) exist in leukemia cells, and we propose their glycolysis regulatory role to maintain the growth of leukemia cells.

## 2. Results

### 2.1. Identification of AML1-ETO Fusion Circular RNAs in AML1-ETO Positive Cell Lines and Primary AML Patients

**Figure 1:**
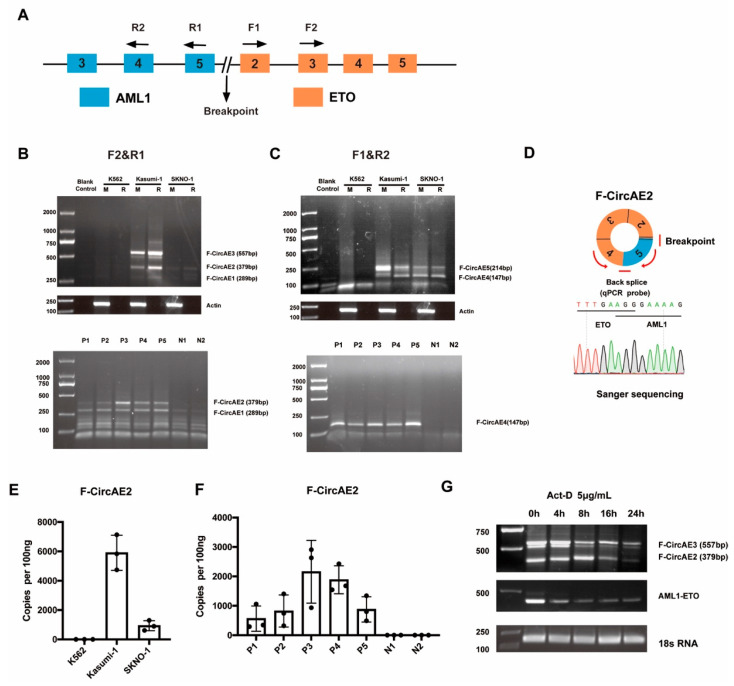
Identification of AML1-ETO fusion circular RNAs in AML1-ETO positive cell lines and primary AML patients: (**A**) Schematic diagram of the *AML1-ETO* translocation and divergent primers used to detect F-CircAEs. Forward primers are located at exon 2 (F1) and exon 3 (F2) of *ETO*, while reverse primers are located at exon 4 (R2) and exon 5 (R1) of *AML1*; (**B**,**C**) PCR analysis of circRNAs derived from *AML1-ETO*-positive (Kasumi-1, SKNO-1), negative (K562) cell lines (upper panel), and primary AML patient BMMNCs harboring *AML1-ETO* translocation (P1–P5) or healthy donors (N1–N2) (lower panel) using primer F2 and R1 (**B**) and F1 and R2 (**C**). F-CircAE1-3 showed amplified products using F2 and R1, and F-CircAE4-5 were the amplified products using F1 and R2. RNAs from cell lines were RNaseR digested (R) or mock-digested (M). RNAs from patients or donors were RNaseR digested; (**D**) Construction of F-CircAE2 and sequence of back-splice site identified by Sanger sequencing; (**E**,**F**) Absolute quantification for F-CircAE2 was conducted by real-time PCR using a TaqMan probe spanning the back-splice site and a pair of divergent primers. Data are presented as mean ± SD (n = 3); (**G**) F-CircAE2 expression after Actinomycin D treatment using PCR.

One of the primary mechanisms for circRNAs biogenesis is the presence of reverse complementary Alu repeats, which help to close the 3′-end of an exon to the 5′-end of the upstream exon [4,20]. Several reverse complementary Alu repeats exist in the intron sequence near the *AML1-ETO* breakpoint (Figure S1) [21], from which it was predicted that the *AML1-ETO* transcript has the potential to form *AML1-ETO*-related fusion circular RNAs (F-CircAEs). Divergent primers were designed near the translocation breakpoint (Figure 1A), and a PCR assay was used to identify F-CircAEs in AML1-ETO positive Kasumi-1 and SKNO-1 cell lines. The K562 cell line was used as a negative control. Cells were treated as described in the Methods section. Several F-CircAEs were amplified using primers F1 and R2 or F2 and R1 in AML1-ETO-positive cell lines, while no detectable F-CircAEs were obtained in K562 cells (Figure 1B,C, upper panel). To further validate that these PCR products were indicative of real circRNAs, RNaseR treatment was conducted, and the amplified products of F-CircAEs were still detectable in AML1-ETO-positive cell lines (Figure 1B,C, upper panel).

Furthermore, F-CircAEs were also identified in bone marrow mononuclear cells (BMMNCs) from adult AML patients harboring *AML1-ETO* (Figure S2 and Figure 1B,C, lower panel). At the same time, no F-CircAE was obtained from normal donor BMMNCs (Figure 1B,C, lower panel). Subsequently, Sanger sequencing of these amplified circular products revealed the head-to-tail sites of F-CircAEs, as shown in Figure S3. These results indicated the existence of F-CircAEs in AML1-ETO cell lines and primary AML patient cells.

As shown in Figure 1B and Figure S3, F-CircAE2 formed by the back-splicing of the AML1 exon 5 and ETO exon 2–4 (Figure 1D) was expressed in both AML1-ETO cell lines and patient BMMNCs. To identify the absolute quantification of F-CircAE2, we performed quantitative real-time PCR analysis using a TaqMan probe, which could recognize the back-splice site of F-CircAE2. The expression level of F-CirsAE2 was significantly higher in the Kasumi-1 cell line and AML1-ETO-positive patient BMMNCs, while the expression level in the SKNO-1 cell line was relatively low (Figure 1E,F). Furthermore, F-CircAE2 and F-CircAE3 were more stable than the *AML1-ETO* linear transcript after exposure to 5 μg/mL actinomycin D at the indicated time points (Figure 1G) [22].

### 2.2. Over-Expression of F-CircAE2 Promoted the Proliferation of NIH3T3 Cells In Vitro

**Figure 2:**
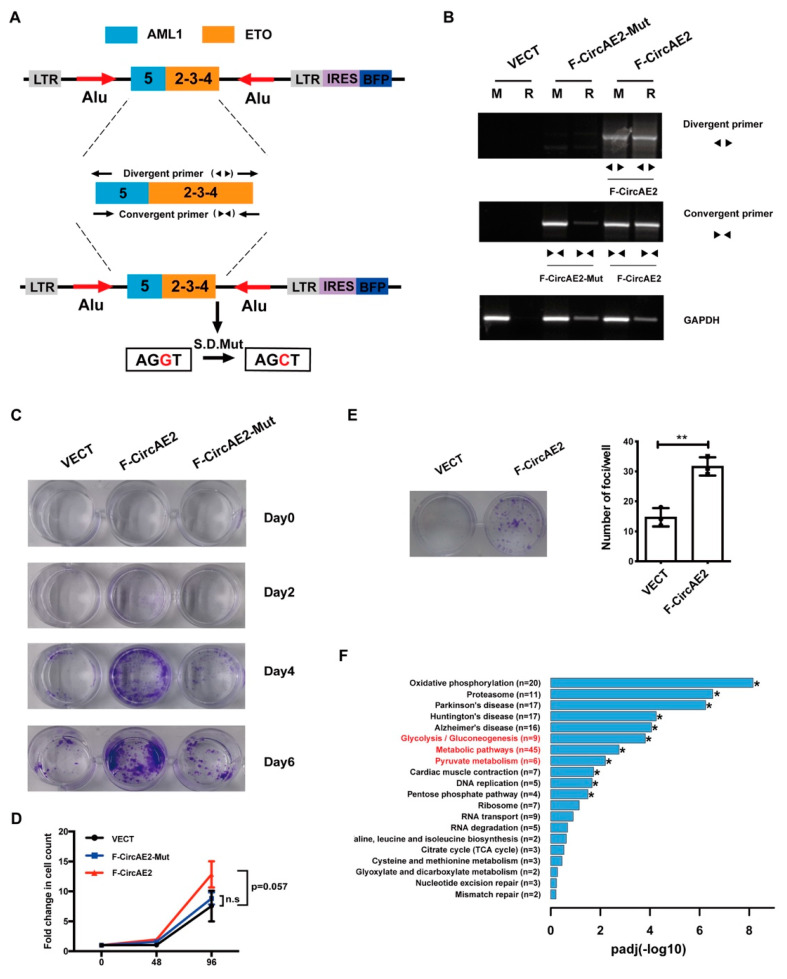
Over-expression of F-CircAE2 promoted proliferation of NIH3T3 cells in vitro: (**A**) Schematic diagram of F-CircAE2 and F-CircAE2 mutant expression retrovirus structure; (**B**) Over-expression of F-CircAE2 was detected using divergent and convergent primers. The F-CircAE-Mut expression can only be detected using the convergent primers. GAPDH was used as a control. R, RNaseR digested. M, mock digested; (**C**) NIH3T3 cells transduced with vector, F-CircAE2 or F-CircAE2-Mut were detected by crystal violet staining at the indicated time; (**D**) The growth of NIH3T3 cells was measured by cell count. Data are presented as mean ± SD (n = 3); (**E**) Representative image showing NIH3T3 foci formation in cell culture dishes (left panel). The right panel shows the number of foci. Data are presented as mean ± SD (n = 3); ** *p* < 0.01; (**F**) Enrichment analysis of up-regulated genes in the F-CircAE2 group, compared with that in the VECT group. Red fonts indicate pathways related to metabolism. * *p* < 0.05, n = 3.

To explore the function of F-CircAEs, we transfected the retroviral expression vector of F-CircAE2 into NIH3T3 cells (F-CircAE2-NIH3T3), using an empty vector (VECT) as a control (Figure 2A). Over-expression of F-CircAE2 (Figure 2B) significantly increased NIH3T3 cell viability and proliferation (Figure 2C,D). The foci formation assays also showed that F-CircAE2-NIH3T3 cells had greatly enhanced foci formation capacity (Figure 2E).

Furthermore, the F-CircAE2-Mut vector was constructed by mutating the splicing donor site of F-CircAE2 from G to C (Table S2), as shown in Figure 2A [20]. As a result, the F-CircAE2, a circularized form of pre-mRNA, failed to be over-expressed in F-CircAE2-Mut-NIH3T3 cells (Figure 2B). Subsequent experiments indicated that F-CircAE2-Mut had a minimal effect on cell viability and proliferation (Figure 2C,D). The findings above revealed that F-CircAE2 in a circular form, rather than linear structure, conferred a selective advantage for cell proliferation independently of AML1-ETO linear transcript and fusion protein.

To determine how F-CircAE2 is involved in the proliferation of NIH3T3 cells, RNA-seq analysis was performed. There were 584 differentially expressed genes (DEGs) between F-CircAE2 and the VECT group, of which 268 were up-regulated in the F-CircAE2 group (Table S6). The up-regulated genes were mapped to the Kyoto Encyclopedia of Genes and Genomes (KEGG) pathway database. The results revealed that the oxidative phosphorylation, glycolysis, metabolic pathways, and pyruvate pathways were closely related to F-CircAE2 expression (Figure 2F and Figure S4, and Table S7), indicating that F-CircAE2 might alter the metabolism of NIH3T3 cells to promote proliferation.

### 2.3. F-CircAE2 Promoted Tumorigenesis of NIH3T3 Cells In Vivo

**Figure 3:**
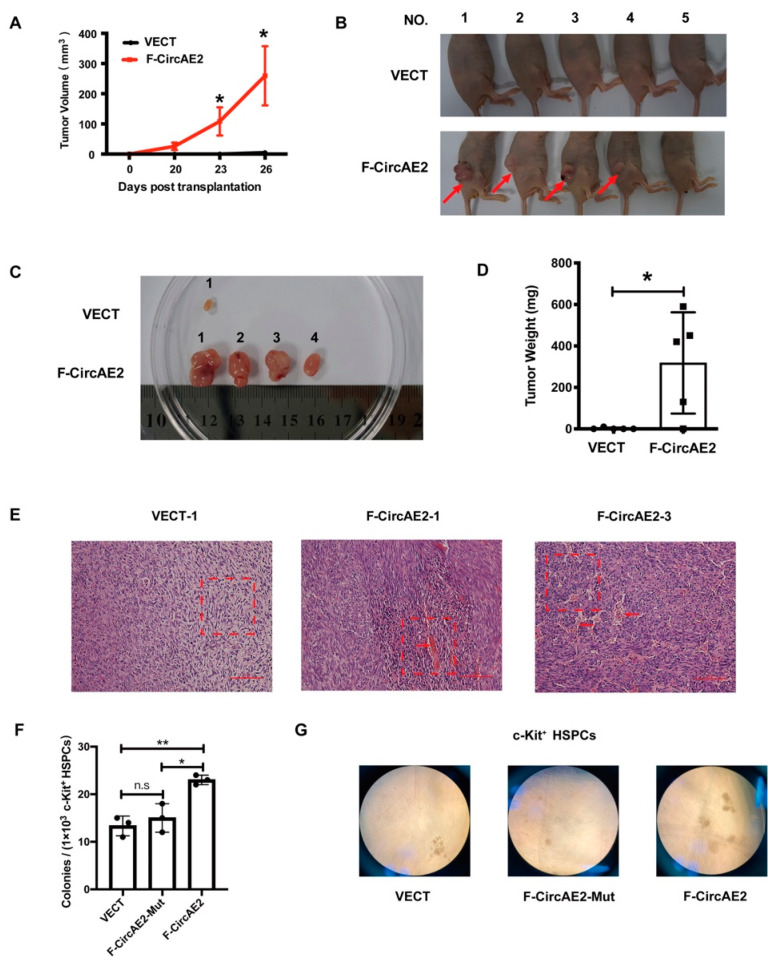
F-CircAE promoted tumorigenesis of NIH3T3 cells in vivo: (**A**) NIH3T3 cells expressing F-CircAE2 or empty vector (VECT) were subcutaneously injected into four-week-old nude mice. Tumor size was measured every three days from when the subcutaneous tumors were visible. * *p* < 0.05; (**B**) Nude mice were sacrificed 26 days after subcutaneous transplantation, and tumor tissues were removed for histological analysis. Red arrows point to the position of subcutaneous tumors; (**C**,**D**) Tumors were dissected from nude mice (**C**), and the tumor weight was measured (**D**). * *p* < 0.05; (**E**) Hematoxylin and eosin (H&E) staining of tumor mass (magnification, 400×). The oval cells (F-CircAE2 group) and fibroblasts (control group) are shown with red dotted frames, highlighting vascularization with arrows; (**F**) The colonies of c-Kit^+^ HSPCs were counted under a microscope. Data are presented as mean ± SD (n = 3); * *p* < 0.05, ** *p* < 0.01; (**G**) Morphology of representative colonies.

The findings above indicated that over-expression of F-CircAE2 promoted NIH3T3 cell proliferation and cellular transformation. Next, we used nude mice to investigate the role of F-CircAE2 in malignant transformation in vivo. NIH3T3 cells stably expressing F-CircAE2 or VECT were injected subcutaneously into nude mice, and the mass growth at the injection site was dynamically monitored. As shown in Figure 3A, the tumor volume in the F-CircAE2 group gradually increased, while no macroscopic tumor appeared in the control group. The mice were humanely sacrificed on day 26 after inoculation, and tumor masses resected from the F-CircAE2 group were much larger and heavier (Figure 3B–D) than the control group. Meanwhile, active angiogenesis was observed on the surface of the tumor **(**Figure 3B,C). H&E staining of tumor masses from the F-CircAE2 group showed visible vascularization, and the cells in which were oval, while the cells in the control group were fibroblasts (Figure 3E). These data suggested that F-CircAE2 also stimulated tumor formation in nude mice.

### 2.4. F-CircAE2 Increased the Clonogenic Ability of c-Kit^+^ HSPCs

AML1-ETO interferes with hematopoietic differentiation in the hematopoietic stem and progenitor cells (HSPCs). F-CircAE2 was derived from the AML1-ETO fusion gene, and how it influences the behaviors of HSPCs was unknown. We over-expressed F-CircAE2 in c-Kit^+^ HSPCs of mice with a retroviral vector (detailed in the Supplemental Methods section). The BFP^+^ cells expressing F-CircAE2 were sorted by flow cytometry (Figure S5). A colony formation assay was performed to evaluate the clonogenic ability. Compared to the VECT or F-CrcAE2-Mut group, HSPCs expressing F-CircAE2 produced more colonies (Figure 3F,G). These data indicated that F-CircAE2 promoted the colony formation ability of HSPCs.

### 2.5. Endogenous F-CircAEs Expression Was Essential for the Maintenance of AML1-ETO-Positive Leukemia Cells

**Figure 4:**
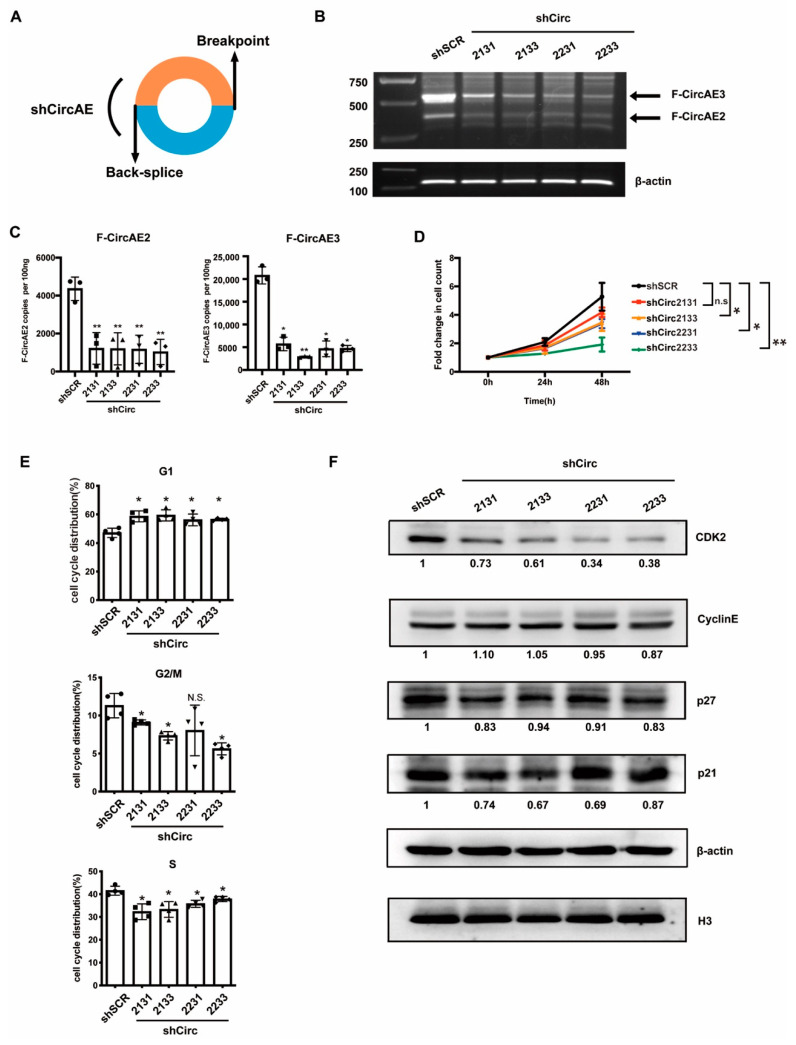
Silencing endogenous F-CircAEs inhibited the proliferation and cell cycle of Kasumi-1 cells: (**A**) Schematic model of the shRNAs. shCircAEs targeting the back-splice junction of F-CircAE2 and F-CircAE3 were used to knock down the F-CircAEs; (**B**,**C**) shCirc21 and shCirc22 were specific for F-CircAE2, shCirc31 and shCirc33 for F-CircAE3 (Figure S6A–F). Different combinations of the two kinds of shRNAs mentioned above (represented as shCirc2131, shCirc2133, shCirc2231, and shCirc2233) were used to knock down the expression of F-CircAE2 and F-CircAE3 simultaneously. The knockdown efficiency was measured by PCR using divergent primers (**B**), and by quantitative real-time PCR using a TaqMan probe spanning the back-splice site (**C**). Data are presented as mean ± SD (n = 3); * *p* < 0.05, ** *p* < 0.01; (**D**) Proliferation of F-CircAEs knockdown or control Kasumi-1 cells was determined using cell count. Data are presented as mean ± SD (n = 3); * *p* < 0.05, ** *p* < 0.01; (**E**,**F**) Cell cycle analysis of F-CircAEs knockdown or control Kasumi-1 cells (**E**). Western blot analysis of CDK2, CyclinE, p27, p21, β-actin, and H3 was used as loading controls. The protein expression levels were normalized to β-actin values, and the relative fold change was obtained by comparing with the control (shSCR) group, as indicated at the bottom for each blot (**F**). Data are presented as mean ± SD (n = 3); * *p* < 0.05.

To examine the function of endogenous F-CircAEs in AML1-ETO-positive cells, we first selected F-CircAE2 (Figure 1B) expressed in both cell lines and patients for targeted silencing studies. The short hairpin RNAs (shCirc21 and shCirc22), which specifically target the back-splice junction of F-CircAE2 (Figure 4A, Table S3), could effectively silence its expression (Figure S6A); the relative levels of the linear form of AML1, ETO, and AML1-ETO in F-CircAE2 knockdown and scramble groups did not show significant differences (Figure S6B). However, individual knockdown of F-CircAE2 was insufficient to impair the proliferation of Kasumi-1 cells (Figure S6C). Next, we disrupted the expression of F-CircAE3 using RNA interference (shCirc31, shCirc33) in Kasumi-1 cells (Figure S6D) and verified that the knockdown of F-CircAE3 did not affect cell proliferation either (Figure S6E).

**Figure 5:**
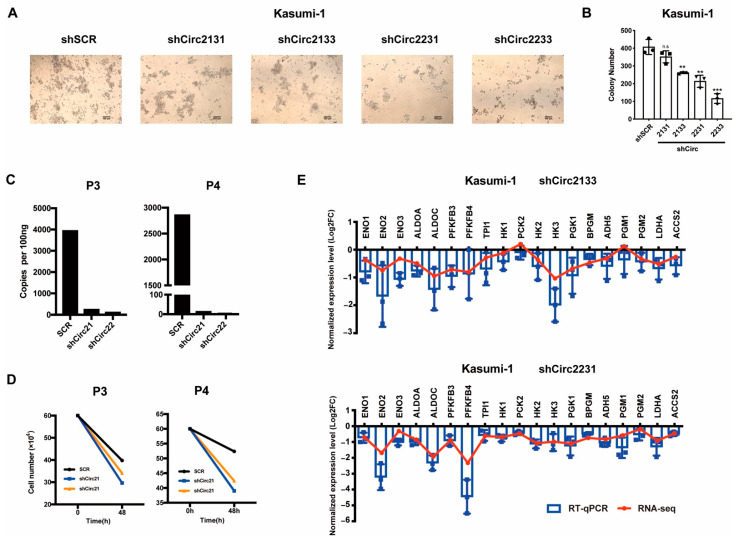
Endogenous F-CircAEs expression is essential for maintaining AML1-ETO-positive leukemia cells: (**A**) Representative morphology of F-CircAEs knockdown or control Kasumi-1 cell colonies (inverted microscope, 10×); (**B**) Colony numbers of Kasumi-1 cells per well. Data are presented as mean ± SD (n = 3); ** *p* < 0.01, *** *p* < 0.001; (**C**) F-CircAE2 knockdown efficiency in patient BMMNCs was measured by quantitative real-time PCR using a TaqMan probe spanning the back-splice site and a pair of divergent primers; (**D**) Cell number of patient BMMNCs was evaluated by cell count after F-CircAE2 knockdown for 48 h (n = 1); (**E**) RNA-Seq expression profiles of glycolysis-related genes that were down-regulated in the F-CircAEs knockdown group were verified by RT-PCR analysis using gene-specific primers. A representative of three independent experiments is shown. Mean ± SD.

We combined the shRNA of each F-CircAE—that is, shCirc2133 (shCirc21 and shCirc31), shCirc2131 (shCirc21 and shCirc31), shCirc2231 (shCirc22 and shCirc31), and shCirc2231 (shCirc22 and shCirc33)—in order to simultaneously reduce the expression of F-CircAE2 and F-CircAE3 (Figure 4B,C). The results demonstrated that the suppression of both F-CircAEs reduced the growth rates of Kasumi-1 cells, compared with that of the shSCR group (Figure 4D). In order to determine whether growth inhibition was caused by cell cycle arrest, PI staining was conducted to examine the cell cycle distribution. As shown in Figure 4E and Figure S7, after disrupting the expression of F-CircAEs, the percentage of Kasumi-1 cells increased in the G1 phase and decreased in S and G2/M phases. We also found that the protein level of CDK2 was significantly down-regulated in the F-CircAEs knockdown groups (Figure 4F). These results indicated that the disruption of F-CircAEs expression could inhibit Kasumi-1 cell growth through G1 phase arrest. We further investigated the effect of F-CircAEs on colony formation capability. Significant reductions in colony size and number of Kasumi-1 cells were observed in the F-CircAEs knockdown groups (Figure 5A,B). Together, the F-CircAEs were essential for the growth and colony formation of Kasumi-1 cells.

To further identify the biological functions of F-CircAE, we used shCirc21 and shCirc22 to target the back-splice site of F-CircAE2 in the BMMNCs of AML1-ETO-positive patients P3 and P4 (Figure 1B and Figure S2). Knockdown of the expression of F-CircAE2 (Figure 5C) inhibited the growth of patient BMMNCs, indicating that F-CircAE2 also participated in the maintenance of primary leukemia cells (Figure 5D).

In summary, F-CircAEs were essential for the survival of *AML1-ETO*-positive cell lines and blast cells. We next compared the transcriptome differences between F-CircAEs knockdown groups and the shSCR group, in order to explore the possible function of endogenous F-CircAEs. An RNA-seq experiment was carried out with F-CircAEs knockdown by shCirc2133, shCirc2231, and shSCR in Kasumi-1 cells. Based on the sequencing reads in RNA-seq (Tables S8 and S9), Gene Set Enrichment Analysis (GSEA) [23] revealed that the glycolysis pathway was enriched following F-CircAEs knockdown in Kasumi-1 cells. Genes encoding proximal components of glycolysis, such as ENO1/2, PGAM1, ALDOA, ALDOC, PGK, and HK1/2/3, were extensively down-regulated (Figure S8, Table S10). These glycolysis genes mentioned above were also significantly reduced by RT-PCR analysis using gene-specific primers (Figure 5E, Table S5). In line with the previous results that F-CircAE2 over-expression was related to the metabolism pathway in NIH3T3 cells, endogenous F-CircAEs in Kasumi-1 may also contribute to the cells’ metabolism alteration and maintain their growth.

### 2.6. F-CircAE2 Bound to Proteins Involved in the Glycolysis Pathway and Promoted Aerobic Glycolysis of Leukemia Cells

The previous results demonstrated that the expression of glycolysis gene sets was positively correlated with elevated F-CircAE2 expression (Figure 2F and Figure 5E), indicating a potential role of F-CircAE2 in regulating glycolysis. Aerobic glycolysis is the main energy-generating pathway of tumor cells, which are characterized by higher glucose consumption and lactate production than normal cells. Glycolysis involves a series of specific steps catalyzed by enzymes that are abnormally expressed in tumor cells [24]. Emerging evidence has indicated that circRNAs interact with proteins and regulate the expression and function of these binding proteins [12,25,26]. Hence, in the following study, we focused on the interactions between F-CircAE2 and these glycolytic enzymes.

**Figure 6:**
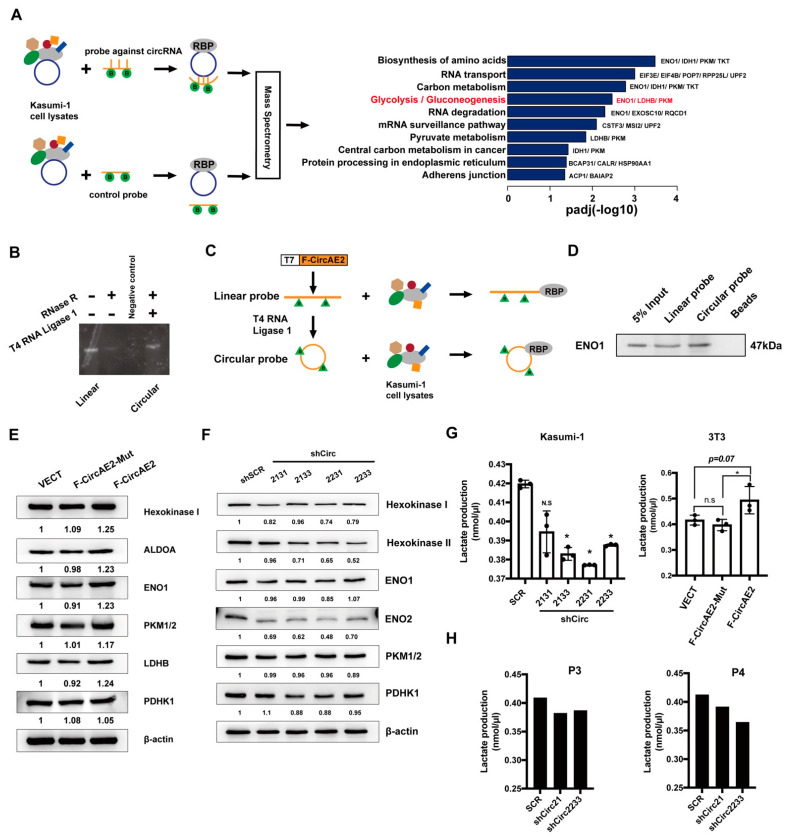
F-CircAE2 binds to proteins involved in the glycolysis pathway and promotes glycolysis: (**A**) Schematic representation of RNA pulldown assay to identify proteins associated with endogenous F-CircAE by mass spectrometry and gene enrichment analysis of F-CircAE2 pulldown proteins. The top 10 enriched pathways are shown; Red font indicates the glycolysis pathway; (**B**) In vitro circularization of F-CircAE2 was analyzed by 1% agarose gel electrophoresis. RNase R treatment was used to validate circular RNA; (**C**) Schematic overview of RNA pulldown assay to identify circRNA–protein interaction with exogenous F-CircAE2 by Western blot; (**D**) Western blot analysis of ENO1 associated with exogenous F-CircAE2, linear form, and negative beads control through RNA pulldown assay. The input was the total protein used for RNA pulldown; (**E**,**F**) Protein levels of glycolytic enzymes were detected by Western blot analysis in F-CircAE2 over-expressed, F-CircAE2-mut over-expressed, or control group NIH3T3 cells (**E**), and F-CircAEs knockdown or scramble group of Kasumi-1 cells (**F**). The protein expression levels were normalized to β-actin values, and the relative fold change was obtained by comparing with the control (shSCR or VECT) group, as indicated at the bottom for each blot. (**G**,**H**) Lactate production in Kasumi-1 cells (**G**, left panel), NIH3T3 cells (**G**, right panel), and BMMCs of AML1-ETO positive patients (**H**) were measured by colorimetric absorbance at OD450 nm. Assays were repeated three times. * *p* < 0.05 (n = 3).

To identify proteins bound to F-CircAE2, we used biotinylated DNA oligo probes against F-CircAE2 at the back-splice site or control probe to incubate with Kasumi-1 cell lysates. Bound proteins in the pulldown materials were washed, eluted, and identified using mass spectrometry (Figure 6A). The data demonstrated that F-CircAE2 is associated with 71 proteins (Figure 6A, Table S11). Then, the ENRICH tool [27], using the Kyoto Encyclopedia of Genes and Genomes (KEGG), was utilized for enrichment analysis of F-CircAE2-associated proteins (Table S12). The data suggested that the binding proteins were enriched in the glycolysis pathway, including LDHB, PKM, and ENO1 (Figure 6A).

To further validate the interaction of F-CircAE2 with LDHB, PKM, and ENO1, an RNA pulldown assay was performed using biotin-labeled F-CircAE2 probes generated by ligation of linear transcript in vitro (Figure 6B,C) [28,29]. The probe was incubated with Kasumi-1 cell lysates, as mentioned in the Methods section (Figure 6C). It was observed that both linear and circularized F-CircAE2 could physically bind to endogenous ENO1 protein (Figure 6D); the effect of different RNA sub-types on glycolysis remained unclear.

To further delineate the influence of F-CircAE2 on glycolysis protein level, it was confirmed, by Western blot, that the protein level of ENO1 increased in the F-CircAE2 over-expressed NIH3T3 cells, compared with that in the VECT and F-CircAE2-Mut groups (Figure 6E). In agreement with the enhanced ENO1 level, the expression of several glycolytic enzymes, such as Hexokinase I, ALDOA, LDHB, and PKM1/2, was also up-regulated in the F-circAE2 group (Figure 6E). Similarly, with F-CircAE2 knockdown in Kasumi-1 cells, the expression levels of glycolytic enzymes (Hexokinase I, Hexokinase II, ENO2, and PDHK1) were reduced (Figure 6F). The results indicated that, although both the linear and circular forms of F-circAE2 could interact with ENO-1, only F-circAE2 enhanced the expression of glycolytic enzymes.

Consistently, we further examined whether the interaction between F-CircAE2 and ENO1 could affect the glycolysis process in cells. It was observed that the knockdown of F-CircAEs reduced lactate production in Kasumi-1 cells (Figure 6G, left panel), while the over-expression of F-CircAE2 increased lactate production in NIH3T3 cells (Figure 6G, right panel). In F-CircAE2 knockdown patient BMMNCs, lactate production was also decreased. These results further support the idea that F-CircAE2 binds with ENO-1 and up-regulates glycolysis to maintain the growth of leukemia cells.

## 3. Discussion

Recent advances have demonstrated that circRNAs are of great significance for the diagnosis, treatment, and prognosis of AML [30]. Microarray and bioinformatics analysis indicate that circRNAs expressed in a leukemia-specific manner compared to healthy control samples [16,31,32]. Moreover, their expression level is associated with leukemia-free and overall survival, indicating their potential use in the diagnosis and prognostication of AML [32]. Emerging evidence has also shown that circRNAs play biological roles in leukemogenesis. CircRNF220 was specifically upregulated in pediatric AML and operated in AML progression through the circRNF220-miR-30a axis [33]. circMYBL2 was reported to recruit PTBP1 to regulate FLT3 translation and promote FLT3-ITD AML progression [34].

The occurrence of AML is often associated with chromosomal translocations. How chromosomal translocation influences the production and biological function of circRNAs have been less studied. A few pioneer studies have exemplified fusion-circRNAs (f-circRNAs) derived from tumor-associated chromosomal translocations, such as *PML-RARα*, *MLL-AF9*, *EWSR1/F LI1*, and *EML4/ALK1* [20,35,36], and these f-circRNAs were circularized by transcribed exons of distinct genes affected by the translocations. In these previous studies, f-circPR [20] and f-circM9 (from *PML-RARα* and *MLL-AF9*, respectively) have been reported to be crucial in promoting leukemia progression and conferring resistance to therapy. Another circular RNA, F-circEA-2a, derived from the EML4-ALK fusion gene, has also been shown to promote cell migration and invasion in non-small cell lung cancer. *AML1-ETO* is one of the most common chromosomal rearrangements in AML; however, the existence of f-circRNAs derived from it had not been previously determined.

Therefore, for the first time, we identified several f-circRNAs derived from *AML1-ETO* in leukemia cell lines (Kasumi-1, SKNO-1) and patient BMMNCs, which we named F-CircAEs (Figure 1). RNaseR treatment, head-to-tail site verification, and absolute quantification of expression using specific probes further validated that they were true circRNAs (Figure 1). The alternative formation and competition of reverse complementary Alu repeats in the intron sequence near the AML1-ETO breakpoint can lead to alternative circularization and regulate the circularization efficiency. In addition, the expression level of F-CircAE is positively correlated with the expression of the AML1-ETO fusion gene. The above factors resulted in multiple f-circRNA transcripts produced from the AML1-ETO fusion gene with different expression levels [4].

We then explored the functional roles of F-CircAEs, in order to obtain new insight into f-circRNAs. F-CircAE2, expressed in both *AML1-ETO*-positive cell lines and the primary leukemia cells, was used for the functional study (Figure 1). Elevated growth and enhanced foci formation were observed in F-CircAE2 over-expressed NIH3T3 cells (Figure 2). In vivo, the subcutaneous inoculation of F-CircAE2-NIH3T3 cells into nude mice led to the development of tumors (Figure 3). These findings demonstrated that F-CircAE2 could promote tumor progression independently of fusion genes and proteins.

To further determine whether the endogenous F-CircAEs were functional, F-CircAE2 and F-CircAE3 were knocked down simultaneously in Kasumi-1 cells. The results demonstrated that the silencing of the two F-CircAEs reduced the growth rate of Kasumi-1 cells and induced G1 cell cycle arrest (Figure 4). As for the BMMNCs of *AML1-ETO*-positive patients, the knockdown of F-CircAE2 also decreased their growth.

These results demonstrated that F-CircAEs are essential for the maintenance of leukemia cells. Although the expression and function of f-circRNAs have been investigated in a few earlier studies, the specific pathogenic mechanism still needs to be studied. In this study, we performed RNA-seq analysis to reveal the global view of the distinguished transcriptome profiles in F-CircAE knockdown Kasumi-1 cells and over-expressed NIH3T3 cells. The results indicated that the gene set of glycolysis was down-regulated upon F-CircAE2 and F-CircAE3 depletion and up-regulated under F-CircAE2 over-expression (Figure 2 and Figure 5). These studies provide the possibility of exploring the relationship between F-CircAE2 and glycolysis.

Current knowledge suggests circRNAs can function as sponges or decoys for proteins in cancer development and progression. Some circRNAs have been reported as being involved in the initiation and development of tumors, through binding proteins associated with cell proliferation and apoptosis. Circ-Foxo3 was shown to interact with MDM2, p53, CDK2, and p21, resulting in cell cycle arrest [12,26], while Circ-CTNNB1 was reported to promote cancer progression by interacting with DDX3 to transactivate YY1 [37]. A series of catalytic enzymes regulate the glycolysis pathway; as such, we speculated that F-CircAE2 could interact with glycolysis protein.

In the presence of oxygen, most tumor cells produce ATP through the aerobic glycolytic pathway to sustain their unlimited proliferation [38]. Aerobic glycolysis is a complex biochemical process, in which glucose is carried into the cell and eventually converted into lactate. This process is regulated by a series of catalytic enzymes, such as hexokinases (HKs), aldolase (ALDO), phosphoglycerate mutase (PGM), enolase (ENO), lactate dehydrogenase (LDH), and so on. Furthermore, glycolytic genes are typically expressed abnormally in tumor cells [39,40], leading to tumor progression and relapse through enhancing aerobic glycolysis [41]. In leukemia cells, there are also metabolic alterations that increase the glycolysis rate and produce excessive lactate [42,43,44]. As circRNAs mainly function as sponges or decoys for proteins, it was plausible to hypothesize that F-CircAE2 might be involved in the glycolysis pathway by interacting with glycolytic enzymes and enhancing glycolysis to maintain the proliferation of leukemia cells.

We uncovered direct circRNA–protein binding through RNA pulldown assay between F-CircAE2 and ENO1. Our bioinformatic and functional studies further elucidated that the protein and mRNA levels of ENO1 and other glycolytic enzymes were up-regulated by F-CircAE2 expression (Figure 2, Figure 5 and Figure 6). Lactate production was elevated with F-CircAE2 expression, confirming that F-CircAE2 could regulate the glycolytic pathway through ENO1. However, more studies are needed to fully and deeply elucidate its biological functions.

Although some studies have reported that circRNAs play regulatory functions on their host genes or proteins [45], F-CircAEs appeared to have no regulatory effect on AML1-ETO in our research, as inhibiting the expression of F-CircAE2 did not affect the mRNA level of *AML1-ETO* in our study. Furthermore, the RNA pulldown assay did not indicate an interaction between F-CircAE2 and AML1-ETO fusion protein. These findings suggest that, although F-CircAE2 is derived from *AML1-ETO*, it can play a functional role independently of its linear transcript and fusion protein, consistent with the independent oncogenic functions of other f-circRNAs, such as f-circPR [20], f-circM9 [20], and F-circEA-2a [36].

Given that F-CircAEs exhibit leukemia cell (AML1-ETO-positive)-specific expression, they can potentially serve as effective therapeutic targets. In our research, an RNA interference (RNAi)-based strategy was used to knock down the expression of F-CircAEs in vitro. This strategy has many limitations for developing therapy due to its instability, immune system activation, off-target effects, and low intracellular content [46,47,48,49]. Several recent developments, including the delivery system (nanoparticle- and exosome-mediated) [50,51], and knockdown (or knockout) system (CRISPR/Cas9 and CRISPR/Cas13 technologies) [52,53], have been used to develop circRNA-based therapeutics. Therefore, there are still many obstacles to circRNA-based therapeutics [48]. Further investigations are needed to advance the clinical potential of F-CircAE-based therapeutics.

## 4. Materials and Methods

### 4.1. Cells and Culture Conditions, Actinomycin D Treatment

Kasumi-1, SKNO-1, and K562 cell lines were routinely maintained in RPMI-1640 medium (with 2 g/L or 4.5 g/L D-Glucose) supplemented with 20%, 20%, and 10% fetal bovine serum (FBS, Invitrogen, USA), respectively. SKNO-1 cells were supplemented with 10 ng/mL recombinant human granulocyte-macrophage colony-stimulating factor. NIH3T3 cells were maintained in Dulbecco’s modified eagle medium supplemented with 10% FBS.

Bone marrow mononuclear cells (BMMNCs) were obtained from five adult patients with AML, and *AML1-ETO* fusion gene expression was analyzed using RT-PCR (Figure S1). Two healthy donors’ BMMNCs were used as a negative control. All patients and healthy donors enrolled in the Institute of Hematology and Blood Diseases Hospital, Chinese Academy of Medical Sciences and Peking Union Medical College (CAMS and PUMC) and gave informed consent. BMMNCs were maintained in IMDM with 15% FBS, 100 ng/mL rhIL-3, 100 ng/mL rhCSF, and 50 ng/mL rhTPO.

A concentration of 5 μg/mL actinomycin D was added to the Kasumi-1 cell culture medium, and cells were harvested at 0, 4, 8, 16, and 24 h. Then the cells were harvested for the following experiments.

### 4.2. RNA Preparation, RNase-R Treatment, and PCR

Total RNA from cell lysates was extracted using Raisa Plus (Takara, Shiga, Japan) and treated for 15 min at 37℃ using RNase-R (Epicentre, Madison, USA) 2 U/μg according to the manufacturer’s instructions. cDNA was synthesized using a reverse transcription kit (Invitrogen, CA, USA) with random primers. Then the cDNA was performed for PCR reaction using HotStarTaq Master Mix (QIAGEN, Hilden, Germany) for up to 40 or 45 cycles. Divergent primers’ sequences were provided in Table S1. The products of PCR were electrophoresed in 1.5% agarose gels, stained with ethidium bromide, observed under UV light, and purified using Agarose Gel DNA Extraction Kit (Takara, Shiga, Japan). The DNA fragment was ligated into a pMD19 T-simple vector and sequenced using Sanger Sequencing.

### 4.3. F-CircAE2 and F-CircAE3 Copy Number Measurement

The divergent primer was used to amplify the sequence containing the back-splicing site of F-CircAE2 and F-CircAE3, and the PCR products were cloned into the pMD-19 T vector. The plasmid as the template for RT-PCR was linearized and serially diluted to generate a standard curve. cDNA synthesized as mentioned above from 2000 ng of RNA, 100 ng RNA of each sample was used for RT-PCR using 2 × TransTaq-T PCR SuperMix (Transgene, Beijing, China) in a QuantStudio real-time PCR system (ThermoFisher, CA, USA). Copy number was calculated according to the standard curve. The primers and probes are listed in Supplementary Table S1.

### 4.4. Proliferation Assay

Kasumi-1 and NIH3T3 cells were seeded in a 6-well plate or 12-well plate at a density of 1 × 10^6^ or 1 × 10^4^ cells/well. BMMNCs were seeded in a 12-well at a density of 6 × 10^5^ cells/well. After incubation for the indicated times, the cells were collected and counted with a hemocytometer after staining with trypan blue.

### 4.5. NIH3T3 Growth and Foci Formation Assays, and Crystal Violet Staining

For growth detection, NIH3T3 cells were seeded in 12-well plates at a concentration of 1 × 10^4^ per well. For the foci formation assay, cells were seeded in 12-well plates at a concentration of 5 × 10^3^ cells per well for 10 days. Cells were fixed with 4% paraformaldehyde and stained with crystal violet at indicated days.

### 4.6. Tumorigenicity in Immunodeficient Mice

Four-week-old female nude mice were obtained from the Institute of Laboratory Animal Sciences (CAMS and PUMC, Beijing, China) and irradiated at a 2.0 Gy dose. F-CircAE-expressing or empty vector-expressing control NIH3T3 cells were suspended in phosphate-buffered saline (PBS), and 1 × 10^6^ cells were injected subcutaneously (n = 5 per group). Tumor volumes were dynamically measured using the formula: (π/6) × (length × width^2^). Mice were sacrificed 26 days after inoculation. The tumor masses were excised, weighed, and then fixed in formalin and stained with hematoxylin and eosin. All animal experiments were approved by the Institutional Animal Care and Use Committee of CAMS and PUMC.

### 4.7. RNA Pulldown Assay

According to the instructions, the RNA pulldown assay was performed with Pierce Magnetic RNA-Protein Pull-Down Kit (Thermo Fisher, CA, USA). In brief, first, 10^7^ cells were collected and washed in ice-cold PBS. The cell pellets were lysed in 1 mL IP lysis buffer and centrifuged at 12,000× *g* for 15 min at 4 °C to collect the supernatant. Second, 50 μL washed streptavidin magnetic beads incubated with 5 μg biotinylated DNA oligo probes against circRNA at the back-splice site or control probe for 30 min at room temperature with agitation (The sequences of probes were shown in Table S4). Then, probe-coated beads were incubated with 500μL cell lysis supernatant for 1 h. The beads were washed briefly with wash buffer five times and eluted. The bound proteins in the pulldown materials were resolved in gradient gel electrophoresis and then digested using trypsin tryptic digestion through a modified Filter-Aided Sample Preparation protocol (FASP). The capillary high-performance liquid chromatography method was used to separate the peptides. Tandem mass spectrometry was used for protein sequencing following the manufacturer’s protocol.

Linear type of F-CircAE was in vitro transcribed using Biotin RNA Labeling Mix (Roche, Basel, Switzerland) and T7 RNA polymerase (Roche, Basel, Switzerland), incubated with PCR product of F-CircAE. Then the T4 RNA ligase 1 (New England Biolabs, Massachusetts, USA) assisted circularization of a linear type of F-CircAE. The lysates of Kasumi-1 cells were incubated with the biotinylated molecule. The washed Dynabeads^TM^ MyOne^TM^ magnetic beads (Invitrogen, CA, USA) were then coated with the biotinylated RNA at room temperature for 2 h. The beads were washed with CO-IP buffer three times and boiled in the protein-loading buffer for 5 min. The retrieved proteins in the pull-down materials were determined by Western blot.

### 4.8. Statistical Analysis

All data were expressed as the means ± SD. Distribution was tested using the Shapiro–Wilks method. When parameters followed the Gaussian distribution, Student’s *t*-test was used. Data analyses were determined by the Student’s *t*-test for experiments involving only two groups and the Mann–Whitney U test for the comparison of tumor weight and volume using Prism5 (GraphPad Software, La Jolla, CA, USA). *p* values < 0.05 were considered statistically significant. * *p*< 0.05, ** *p*< 0.01, and *** *p*< 0.001 in comparison.

## 5. Conclusions

Given the observations outlined above, we first proved the existence of *AML1-ETO*-related fusion circular RNAs. More importantly, F-CircAEs themselves—that is, without AML1-ETO fusion protein—can promote cell proliferation. The knockdown of endogenous F-CircAEs led to the proliferation inhibition of *AML1-ETO*-positive cells. Our key observation was that F-CircAE2 exerts its biological activity by interacting with glycolytic enzymes and up-regulating the level of glycolysis, which provides further evidence for the molecular mechanism of f-CircRNA. The discovery of F-CircAEs in *AML1-ETO* leukemia constitutes considerable progress in understanding their pathogenic mechanism. F-CircAEs may be used as new molecular markers for disease diagnosis, as well as new targets for the development of novel therapeutic strategies.

## Data Availability

Not applicable.

## Supplementary Materials

The following supporting information can be downloaded at: https://www.mdpi.com/article/10.3390/ijms24010071/s1. Reference [54] is cited in the supplementary materials.
