# Supplementary material for: AML1-ETO-Related Fusion Circular RNAs Contribute to the Proliferation of Leukemia Cells
Source: Int J Mol Sci. 2022 Dec 21;24(1):71. doi: 10.3390/ijms24010071 (PMC9820653; doi:10.3390/ijms24010071)
Supplement: Supplementary file 1 [file ijms-24-00071-s001.zip › supplemental figure sub IJMS.pdf]

Figure S1

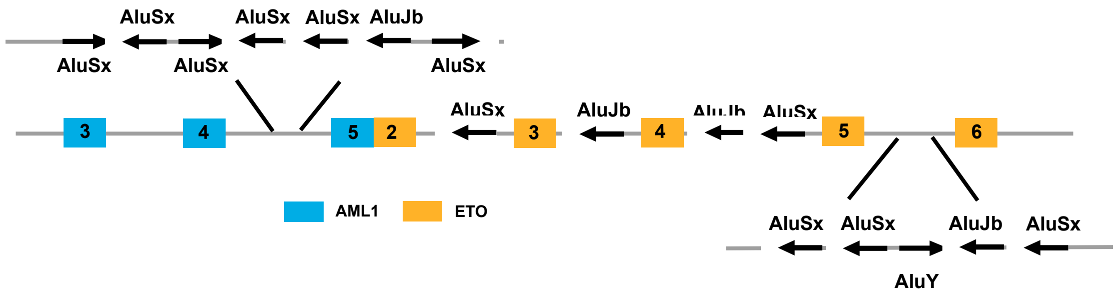

Figure S1 Alu repeats that existed in the intron sequence near the *AML1-ETO* breakpoint were recognized by RepeatMasker (http://www.repeatmasker.org).

Figure S2

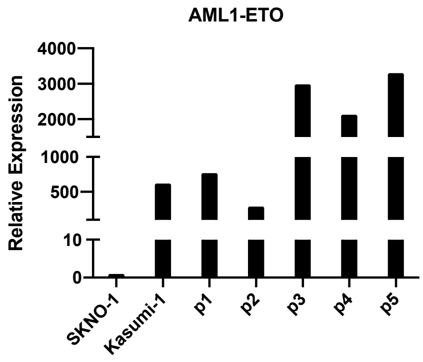

Figure S2 RT-PCR analysis of AML1-ETO expression in Kasumi-1, SKNO-1, and BMMNCs of AML patients.

Figure S3

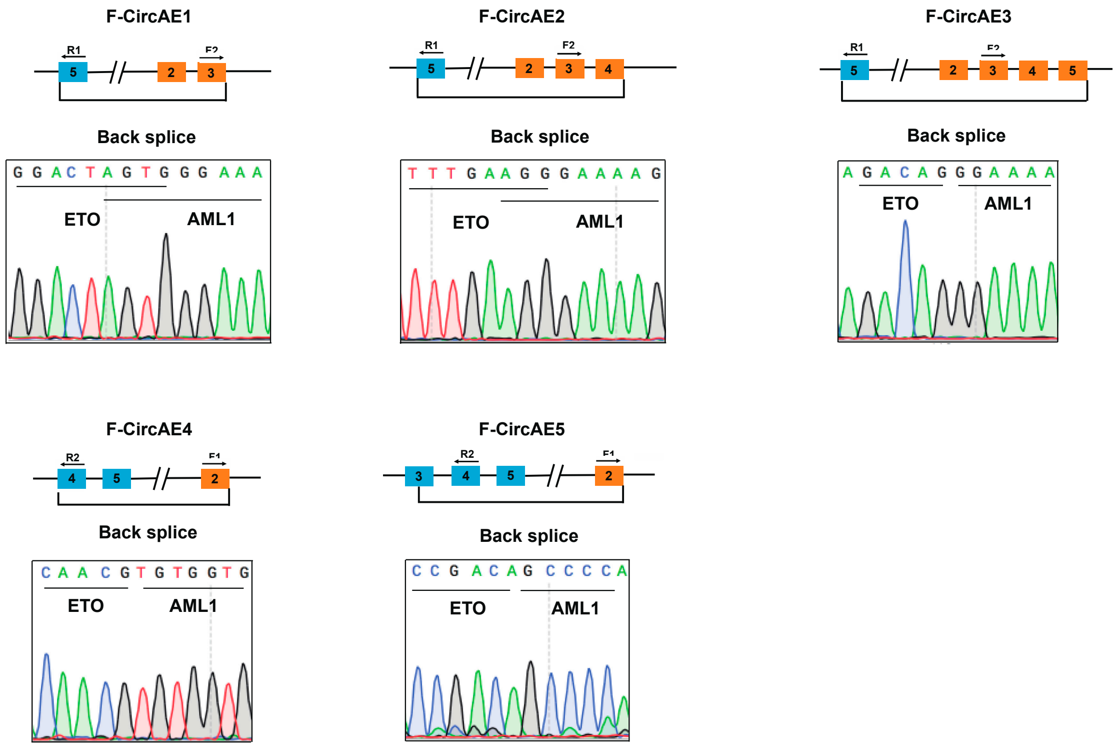

Figure S3 The composition and head-to-tail splicing sites of F-CircAEs were represented. The sequences of F-CircAE2 and F-CircAE3 have been submitted to the GenBank database (MG551955, MG551956).

Figure S4

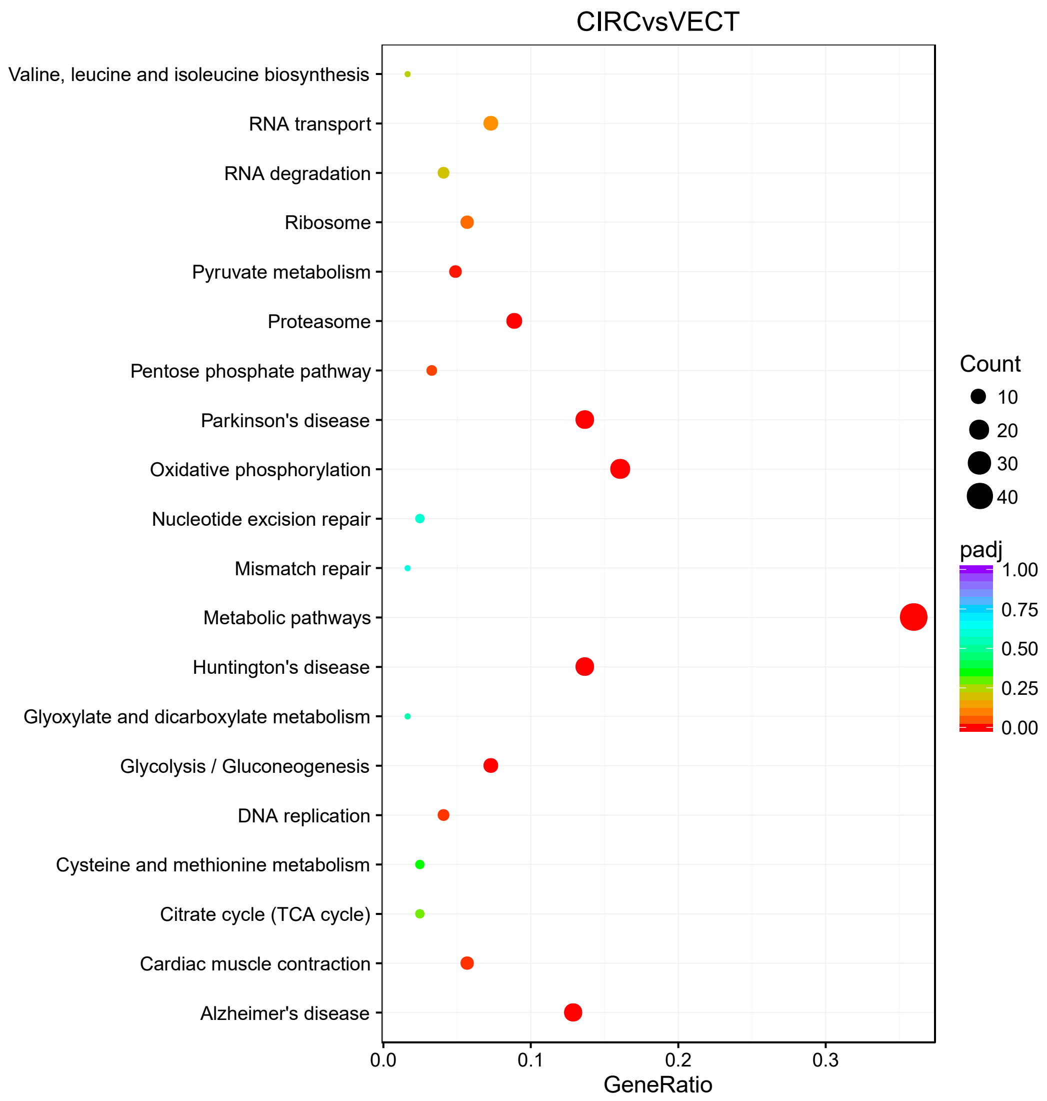

Figure S4 KEGG analysis was performed on the up-regulated DEGs between the F-CircAE2 and the VECT groups in NIH3T3 Cells. The vertical axis represents the top 20 enriched pathways according to the gene counts. The different colors represent the −log10 of adjusted *p* values. The horizontal axis represents the Gene ratio.

Figure S5

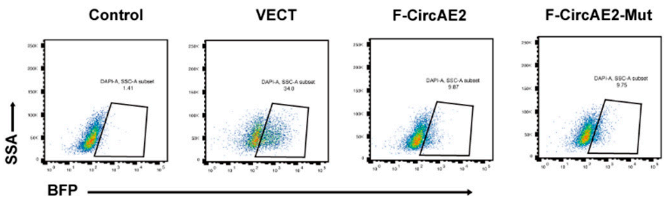

Figure S5 The BFP^+ HSPCs were sorted through flow cytometry.

Figure S6

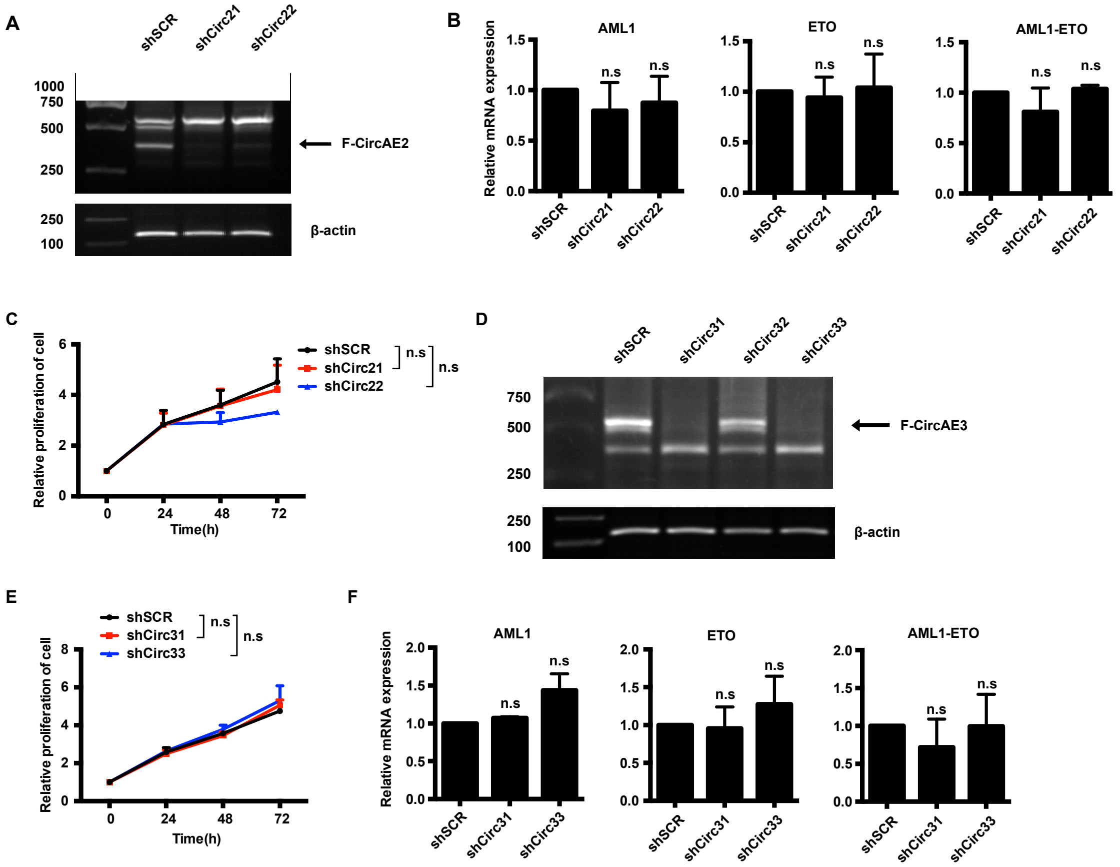

<u>Figure S6</u>

(A) shCirc21 and shCirc22 targeting the back-splice junction of F-CircAE2 efficiently knocked down the expression of F-CircAE2, β-actin was used as control.

(B) RT-PCR analysis of linear transcripts of *AML1*, *ETO,* and *AML1-ETO* using specific primers after F-CircAE2 knockdown.

(C) The proliferation of F-CircAE2 knockdown Kasumi-1 cells showed no significant difference compared with the scramble group.

(D) shCirc31, shCirc32, and shCirc33 were specific for F-CircAE3 knockdown. The knockdown efficiency was measured by PCR using outfacing primers, while β-actin was used as control.

(E) F-CircAE3 knockdown did not affect Kasumi-1 cells proliferation.

(F) RT-PCR analysis of linear transcripts of AML1, ETO, and AML1-ETO using specific primers.

Figure S7

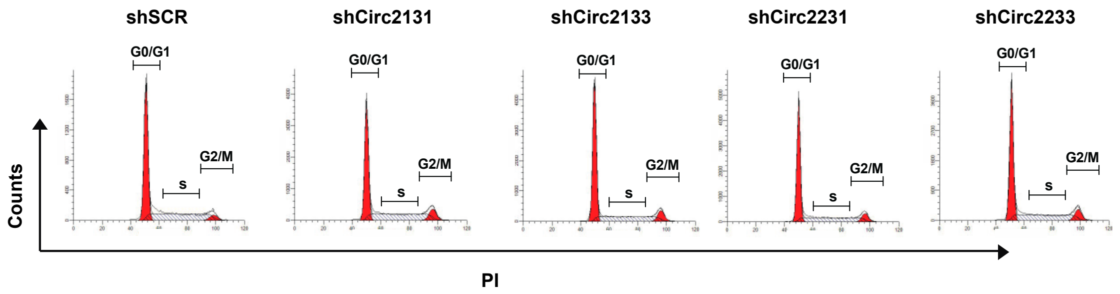

<u>Figure S7</u> Cell cycle distribution was analyzed by flow cytometry using PI staining.

Figure S8

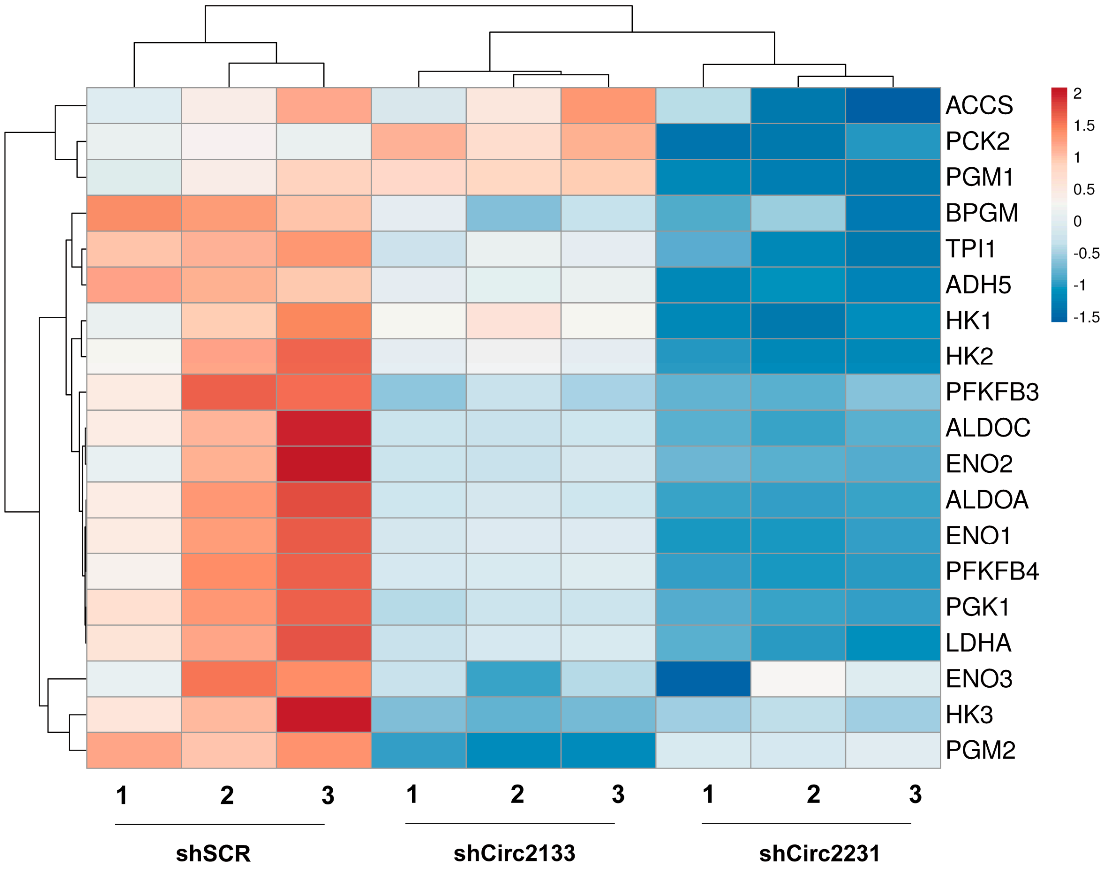

Figure S8 The heatmap of differentially expressed genes in the glycolysis pathway upon F-CircAEs knockdown in Kasumi-1 cells as identified by RNA-seq experiments. Heatmaps were generated using ClustVis (https://biit.cs.ut.ee/clustvis/). The different colors represent the row centered fold change values (blue = lower, white = intermediate, red = higher).
