# Supplementary material for: AML1-ETO-Related Fusion Circular RNAs Contribute to the Proliferation of Leukemia Cells
Source: Int J Mol Sci. 2022 Dec 21;24(1):71. doi: 10.3390/ijms24010071 (PMC9820653; doi:10.3390/ijms24010071)
Supplement: Supplementary file 1 [file ijms-24-00071-s001.zip › Supplemental methods sub.pdf]

Supplemental Methods

Construction of F-CircAE2

Based on the sequence of AML1 and ETO (https://www.ncbi.nlm.nih.gov/pubmed), the Alu elements were found on the website http://www.repeatmasker.org. AluSx3 and AluJb were chosen as the complementary repetitive intronic sequences.

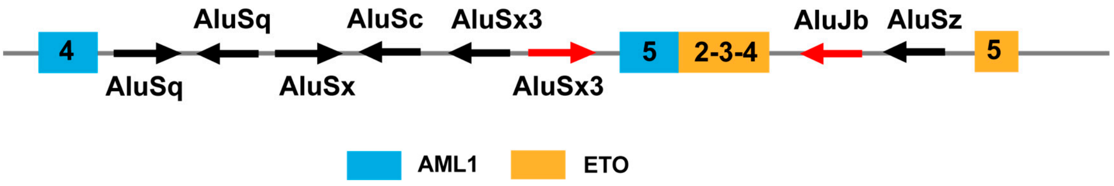

NEBuilder assembly tool (http://nebuilder.neb.com) provided the primer sequences, annealing temperatures, and the sequence of assembled vector.

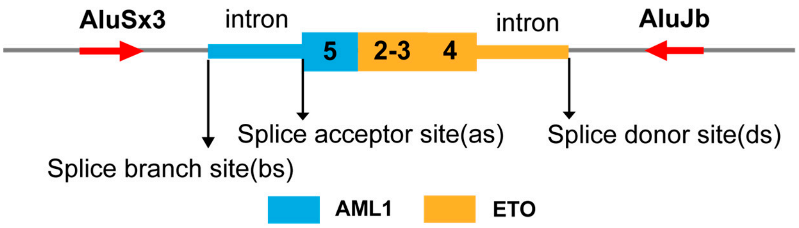

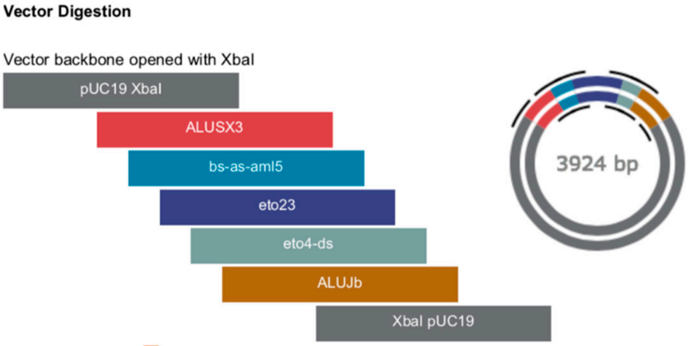

Required Primers

Overlaps	Oligo (Uppercase = gene-specific primer)	Anneals	F/R	3' Tm	3' Ta *	6-Frame
pUC19	gctcggtacccggggatcctTTGGGAGGCCAAGGTGGG	ALUSX3	Fwd	63.6°C	53.7°C	view
bs-as-aml5	atatctgttgTTTTTTTTTTTTTTTTTTTTTTGAGATGGAGTCTTGCACTG	ALUSX3	Rev	58.7°C	53.7°C	view
ALUSX3	aaaaaaaaaaaaCAACAGATATGTTCAGGCCACC	bs-as-aml5	Fwd	58.1°C	53.1°C	view
eto23	tcagtacgatTTCGAGGTTCTCGGGGCC	bs-as-aml5	Rev	63.0°C	53.1°C	view
bs-as-aml5	gaacctcgaaATCGTACTGAGAAGCACTCC	eto23	Fwd	55.9°C	50.9°C	view
eto4-ds	aagtggagttCACTAGTCCCAGAACGAGG	eto23	Rev	56.9°C	50.9°C	view
eto23	gggactagtgAACTCCACTTTGACAATTGAAGAATTTC	eto4-ds	Fwd	55.5°C	50.5°C	view
ALUJb	gaaaaagaaaTGACCTAGCAACAATATGGTTTAAAATACTTTAC	eto4-ds	Rev	56.4°C	50.5°C	view
eto4-ds	tgctaggtcaTTTCTTTTTCTTTCTTTCTTTTTTTTTTTTTTTTTTTTGAAACAAAG	ALUJb	Fwd	55.5°C	50.5°C	view
pUC19	tgcatgcctgcaggtcgactAACACTTCAGGAGGTCAAGG	ALUJb	Rev	56.5°C	50.5°C	view

Plasmid constructs

F-CircAE retroviral expression vector

Gibson Assembly Cloning Kit (New England Biosciences, USA) was used to clone the expression vector of F-CircAE [20]. The intronic and exonic sequences were obtained from genomic DNA and cDNA, respectively, then synthesized and inserted into the pUC19 vector. Supplemental Methods showed a schematic representation of fragment arrangement, vector digestion, and overlapping primers. Then the insertion of assembled DNA fragment was ligated into a retroviral vector MSCV-MCS-IRES-BFP. The empty vector was used as a control.

The F-CircAE-Mut retroviral expression vector

The F-CircAE2-Mut linear transcript was constructed by mutating the splicing donor site of F-CircAE2 from G to C, as shown in Figure 2A, through overlap PCR, using the assembled DNA fragment as a template. Overlapping primers are shown in Table S2. Then the F-CircAE2-Mut fragment was ligated into a retroviral vector MSCV-MCS-IRES-BFP mentioned above.

Establishment of stable cell lines

The short hairpin double-stranded oligo (Table S3) targeting the back-splice junction of the F-CircAE was inserted into the pLKO.1-puro or pLKO.1-GFP lentiviral vector. The successfully constructed vectors were verified by restriction digestion and sequencing. The scramble was used as a control (Table S3). Kasumi-1 cells and BMMNCs from AML1-ETO positive patients were infected with lentivirus carrying pLKO.1-puro or pLKO.1-GFP lentiviral plasmid. Stable gene silencing is selected using the puromycin selectable marker or fluorescent cell sorting.

Retrovirus and lentivirus generation and infection

Retrovirus and lentivirus generation and infection were described before [54].

Colony Formation Assay of c-Kit+ HSPCs.

Six-week-old female C57BL/6 mice were obtained from the Institute of Laboratory Animal Sciences (CAMS & PUMC, China). According to the manufacturer's instructions, the c-Kit+ hematopoietic stem and progenitor cells (HSPCs) from murine bone marrow were purified with anti-c-Kit microbeads (Miltenyi Biotec, USA). The isolated c-Kit$^+$ cells were pre-stimulated with murine IL-3, IL-6, and SCF, followed by infection of retroviral supernatant (MSCV-MCS-IRES-BFP). Then the BFP$^+$ cells were sorted by flow cytometry, and 1×10^3 cells per well were plated into a 48-well plate triplicate in MethoCult M3434 methylcellulose. After a week, colonies (> 40 cells) were counted.

Western blot analysis

Total cellular lysates were obtained with RIPA lysis buffer containing PMSF (Roche, Switzerland) and protease inhibitor cocktail (Roche, Switzerland). The protein samples were run on a 10% SDS-PAGE gel and transferred to a polyvinylidene difluoride membrane. Then the membranes were incubated with cell cycle associated antibodies against CDK2, CyclinE, p27, p21(Cell Signaling Technology, USA) and glycolysis-associated antibodies (Glycolysis Antibody Sampler Kit #8337, Glycolysis II Antibody Sampler Kit #12866, Cell Signaling Technology, USA). Anti-β-actin (Sigma-Aldrich, USA) was used as a loading control. The immunoreactive proteins were visualized using the SuperSignal chemiluminescent detection system (Pierce, USA).
