# Supplementary material for: AML1-ETO-Related Fusion Circular RNAs Contribute to the Proliferation of Leukemia Cells
Source: Int J Mol Sci. 2022 Dec 21;24(1):71. doi: 10.3390/ijms24010071 (PMC9820653; doi:10.3390/ijms24010071)
Supplement: Supplementary file 1 [file ijms-24-00071-s001.zip › Supplemental Table-S1-S5.pdf]

Supplemental Tables

Table S1. Divergent primers sequences for the identification of AML1-ETO-related fusion circular RNAs. Forward primers are located at exon 2 (F1) and exon 3 (F2) of *ETO*, while reverse primers are located at exon 4 (R2) and exon 5 (R1) of *AML1*.

Forward 1 (F1, ETO exon2)	5'-CCTGTGGATGTGAAGACGCA-3'
Forward 2 (F2, ETO exon3)	5'-TCCTACAGCCTTGAATGGCG-3'
Reverse 1 (R1, AML1 exon5)	5'-CTGTGGTAGGTGGCGACTTG-3'
Reverse 2 (R2, AML1 exon4)	5'-ACAGTGACCAGAGTGCCATC-3'

Primer and probe sequences for F-CircAE copy number detection. The F-CircAE2-F-pro, R1 primer set was used to identify F-CircAE2; and F-CircAE3-F-pro, R1 primer set was used to identify F-CircAE3.

F-CircAE2 Probe	5'-FAM-TCCCATTTTTGAAGGGAAAAGCTTCACTCT-MGB-3'
F-CircAE3 Probe	5'-FAM-ACTCCAGACAGGGAAAAGCTTCAC-MGB-3'
F-CircAE2-F-pro	5'-ACTGCAAGAAGCTACTAACTTC-3'
F-CircAE3-F-pro	5'-GCTGCTTCTCGATGTGAACG-3'
R1	5'-CTGTGGTAGGTGGCGACTTG-3'

Table S2. Primer sequences for overlap PCR. The F-CircAE2-Mut linear transcript was constructed by mutating the splicing donor site of F-CircAE2 from G to C, as shown in Figure 2A, through overlap PCR (as shown below), using the assembled DNA fragment as a template. Overlapping primers are shown in Table S2. The red uppercase letters show the mutation bases.

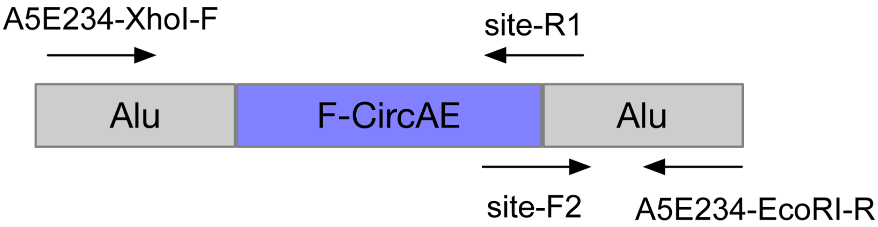

AE2-XhoI-F	5'-CCTGTGGATGTGAAGACGCA-3'
site-R1	5'- GAACTGTGCAATAGCTTCAAAAAT -3'
site-F2	5'- CCCATTTTTGAAGCTATTGCACAG -3'
AE2-EcoRI-R	5'-ACAGTGACCAGAGTGCCATC-3'

Table S3. shRNA sequences targeting back-splice junction of F-CircAEs. shCirc21 and shCirc22 specifically target the F-CircAE2. shCirc31, shCirc32, and shCirc33 target the back-splice junction of F-CircAE3.

shCirc21	sense	TTTGAAGGGAAAAGCTTCACT
	antisense	AGTGAAGCTTTTCCCTTCAAA
shCirc22	sense	TGAAGGGAAAAGCTTCACTCT
	antisense	AGAGTGAAGCTTTTCCCTTCA

shCirc31	sense	AAGCTTTTCCCTGTCTGGAGT
	antisense	ACTCCAGACAGGGAAAAGCTT
shCirc32	sense	GCTTTTCCCTGTCTGGAGTTC
	antisense	GAACTCCAGACAGGGAAAAGC
shCirc33	sense	TGAAGCTTTTCCCTGTCTGGA
	antisense	TCCAGACAGGGAAAAGCTTCA

shSCR	sense	CCTAAGGTTAAGTCGCCCTCG

	antisense	CGAGGGCGACTTAACCTTAGG

Table S4. DNA oligo probe sequence targeting the back-splice site of F-CircAE2. The sequence of the control probe is the antisense sequence.

against F-CircAE2	5'-biotin-aaaCAGAGTGAAGCTTTTCCCTTCAAAAATGGGAT-3’
antisense probe	5'-biotin-aaaATCCCATTTTTGAAGGGAAAAGCTTCACTCTG-3'

Table S5. Primers used for RT-PCR analysis of glycolysis-related mRNA levels in Kasumi-1 cells.

ALDOA F	ATGCCCTACCAATATCCAGCA
ALDOA R	GCTCCCAGTGGACTCATCTG
ALDOB F	GGCAGTTCCGAGAAATCCTCT
ALDOB R	CTCCTTGGTCTAACTTGATTCCC
ALDOC F	ATGCCTCACTCGTACCCAG
ALDOC R	TTTCCACCCCAATTTGGCTCA
PFKFB3 F	ATTGCGGTTTTCGATGCCAC
PFKFB3 R	GCCACAACTGTAGGGTCGT
PFKFB4 F	TCCCCACGGGAATTGACAC
PFKFB4 R	GGGCACACCAATCCAGTTCA
ENO1 F	AAAGCTGGTGCCGTTGAGAA
ENO1 R	GGTTGTGGTAAACCTCTGCTC
ENO2 F	AGCCTCTACGGGCATCTATGA
ENO2 R	TTCTCAGTCCCATCCAACTCC
ENO3 F	GGCTGGTTACCCAGACAAGG
ENO3 R	TCGTACTTCCCATTGCGATAGAA
PGK1 F	TGGACGTTAAAGGGAAGCGG
PGK1 R	GCTCATAAGGACTACCGACTTGG
PGM1 F	CCAAACCGACTGAAGATCCGT
PGM1 R	CATGTTTCGATCCCCATCTCC

PGM2 F	GAGGCAGTGAAACGACTAATAGC
PGM2 R	CTGTCCCAAACTCCATTCGGG
HK1 F	GCTCTCCGATGAAACTCTCATAG
HK1 R	GGACCTTACGAATGTTGGCAA
HK2 F	GAGCCACCACTCACCCTACT
HK2 R	CCAGGCATTCGGCAATGTG
HK3 F	GGACAGGAGCACCCTCATTTC
HK3 R	CCTCCGAATGGCATCTCTCAG
TPI1 F	CTCATCGGCACTCTGAACG
TPI1 R	GCGAAGTCGATATAGGCAGTAGG
PCK2 F	GCCATCATGCCGTAGCATC
PCK2 R	AGCCTCAGTTCCATCACAGAT
ACSS2 F	AAAGGAGCAACTACCAACATCTG
ACSS2 R	GCTGAACTGACACACTTGGAC
BPGM F	TGCTTGGAATAAGGAGAACCGT
BPGM R	CCACAGTTCCGAGCTTCCTC
ADH5 F	ATGGCGAACGAGGTTATCAAG
ADH5 R	CATGTCCCAAGATCACTGGAAAA
LDHA F	ATGGCAACTCTAAAGGATCAGC
LDHA R	CCAACCCCAACAACTGTAATCT

Table S6. List of 584 differentially expressed genes (DEGs) between F-CircAE2 and VECT group in NIH3T3 cells, in which 268 genes were upregulated.

Table S7. The top 11 enriched gene ontology terms from the Kyoto Encyclopedia of Genes and Genomes (KEGG) pathway analysis on up-regulated genes in F-CircAE2-NIH3T3 cells vs control.

Table S8. The table shows transcripts differentially expressed between shCirc2133 and control Kasumi-1 cells.

Table S9. The table shows transcripts differentially expressed between shCirc2231 and control Kasumi-1 cells.

Table S10. The pathways enriched following F-CircAEs knockdown in Kasumi-1 cells by Gene Set Enrichment Analysis (GSEA).

Table S11. The proteins binding with F-CircAE2.

Table S12. The top enriched KEGG pathways of F-CircAE2-associated proteins.
